# Optic Pathway Glioma in Type 1 Neurofibromatosis: Review of Its Pathogenesis, Diagnostic Assessment, and Treatment Recommendations

**DOI:** 10.3390/cancers11111790

**Published:** 2019-11-14

**Authors:** Matteo Cassina, Luisa Frizziero, Enrico Opocher, Raffaele Parrozzani, Ugo Sorrentino, Elisabetta Viscardi, Giacomo Miglionico, Edoardo Midena, Maurizio Clementi, Eva Trevisson

**Affiliations:** 1 Clinical Genetics Unit, Department of Women’s and Children’s Health, University of Padova, 35128 Padova, Italy; ugo.sorrentino@aopd.veneto.it (U.S.);; 2 Istituto di Ricerca Pediatrica—IRP, Città della Speranza, 35127 Padova, Italy; 3 IRCCS-Istituto di Ricovero e Cura a Carattere Scientifico—Fondazione Bietti, 00198 Rome, Italy; lfrizziero@gmail.com (L.F.); edoardo.midena@unipd.it (E.M.); 4 Hematology Oncology Division, Department of Women’s and Children’s Health, University of Padova, 35128 Padova, Italy; enrico.opocher@aopd.veneto.it (E.O.); elisabetta.viscardi@unipd.it (E.V.); 5 Paediatric Oncology, Blood, Cell, and Cancer Department, Great Ormond Street Hospital, London WC1N 3JH, UK; 6 Department of Ophthalmology, University of Padova, 35128 Padova, Italy; raffaele.parrozzani@unipd.it (R.P.); giacomo.miglionico@aopd.veneto.it (G.M.)

**Keywords:** type 1 neurofibromatosis, NF1, optic pathway glioma, brain/orbit MRI, clinical trials

## Abstract

Type 1 neurofibromatosis (NF1) is a dominantly inherited condition predisposing to tumor development. Optic pathway glioma (OPG) is the most frequent central nervous system tumor in children with NF1, affecting approximately 15–20% of patients. The lack of well-established prognostic markers and the wide clinical variability with respect to tumor progression and visual outcome make the clinical management of these tumors challenging, with significant differences among distinct centers. We reviewed published articles on OPG diagnostic protocol, follow-up and treatment in NF1. Cohorts of NF1 children with OPG reported in the literature and patients prospectively collected in our center were analyzed with regard to clinical data, tumor anatomical site, diagnostic workflow, treatment and outcome. In addition, we discussed the recent findings on the pathophysiology of OPG development in NF1. This review provides a comprehensive overview about the clinical management of NF1-associated OPG, focusing on the most recent advances from preclinical studies with genetically engineered models and the ongoing clinical trials.

## 1. Introduction

Type 1 neurofibromatosis (NF1 #MIM 162200) is the most common neurocutaneous disorder, affecting approximately one out of 3000 people worldwide [1]. This autosomal dominant tumor predisposition syndrome is caused by inactivating mutations in *NF1*, a tumor suppressor gene which encodes the RAS inhibitor neurofibromin involved in the regulation of cell growth and survival [2].

This condition is characterized by wide allelic heterogeneity, with more than 3000 different variants reported so far, owing to the high mutational rate of the *NF1* gene and the absence of hotspots [3]. NF1 exhibits an extremely variable clinical expressivity, with most patients manifesting cutaneous and ocular signs, including café-au-lait spots, inguinal and axillary freckling, iris hamartomas and choroidal nodules by 6 years of age. A portion of NF1 patients develop one or more complications, including learning disabilities that affect up to 60% of children [4]. The hallmark of this condition are neurofibromas, benign tumors originating from Schwann cells, which typically arise during adulthood, except for plexiform neurofibromas that are congenital [5].

The predisposition to develop tumors involves also the central nervous system: the glioma of the optic pathway (OPG) is a relatively frequent complication of NF1 affecting around 20% of patients, is mostly observed during childhood [6] and is included in the diagnostic criteria [7].

Although being usually characterized by an indolent course, a variable portion of patients manifests symptoms, mainly vision loss and other ophthalmological symptoms, but also precocious puberty or neurological manifestations [8]. The comprehension of the biology of these tumors has improved significantly over the last few years but challenges still remain: (i) the risk assessment in asymptomatic patients remains demanding, owing to the lack of valid biomarkers and the absence of prospective studies that may help in prognosis definition; (ii) the young age of this unique at-risk population and the learning disabilities that frequently coexist complicate the development of an effective OPG screening; (iii) treatments able to prevent or recover vision loss in patients with OPG are still not available.

In this review we will summarize and discuss the clinical features of OPG, the current diagnostic and therapeutic protocols and the most recent advances on its pathophysiology obtained from preclinical models.

## 2. Optic Pathway Gliomas in NF1

### 2.1. Prevalence, Clinical Features and Natural History of OPG

OPG is the most common central nervous system neoplasia detected in pediatric patients affected by NF1, with an estimated prevalence ranging from 15% to 20% [6,9,10,11,12,13,14,15,16,17]. In the majority of cases, NF1-associated OPGs are classified as WHO grade I pilocytic astrocytomas and only 30–50% of patients show signs or symptoms correlated with the tumor [11,16,17,18,19,20]; in addition, they usually present at a younger age compared to sporadic OPGs in the general population [21] and are characterized by an indolent course, with only one-third of the affected patients requiring a specific treatment [20]. Some studies reported a higher prevalence of OPGs among females [6,10,15,22,23], but several others did not observe such a difference according to sex [16,17,24,25].

A recent study evaluated the prevalence of OPG in an unselected cohort of patients with NF1 followed up in a single NF clinic in Germany between 2003 and 2015; all patients were offered whole-body and head MRIs regardless of the presence of symptoms suggestive of OPG [17]. The authors identified a particularly high prevalence of asymptomatic OPG among children younger than 10 years (approximately 20%), which dropped to 5–10% in the group of patients aged 10–19.9 years; on the other hand, the prevalence of symptomatic OPG was lower than 5% in patients younger than 10 and approximately 5% in those aged 10-19.9 years [17]. The prevalence of asymptomatic OPG in children under 10 years was higher than in other studies, but this may be due to the use of different radiologic criteria for the diagnosis; for example, a T2 hyperintensity of the optic nerve was classified as OPG by Sellmer et al. [17] without taking into consideration its thickness or tortuosity.

Our experience at the NF1 clinic of the University Hospital of Padova (Italy) was also published [6]. We analyzed a cohort of 414 consecutive patients affected by NF1 who were first evaluated before the age of 6 years and without a previous diagnosis of OPG; the inclusion criteria were chosen to avoid bias in patients’ selection and the mean duration of follow-up was 11.9 years. In our clinic, screening MRI is not systematically performed in all patients. A total of 52 patients (12.6%) developed OPG during their follow-up, with an estimated cumulative incidence of 15.4% at the age of 15 (Kaplan–Meier analysis). In particular, 25 children were diagnosed with OPG after brain and orbit MRI was performed because of the presence of signs and/or symptoms suggestive of OPG; the remaining 26 did not show symptoms related to OPG at the diagnosis and underwent brain imaging because of other clinical indications (including macrocephaly, developmental delay, headache, seizures, plexiform/orbital neurofibromas of the face/neck, brain ischemia) or for screening surveillance in other hospitals.

**Figure 1:**
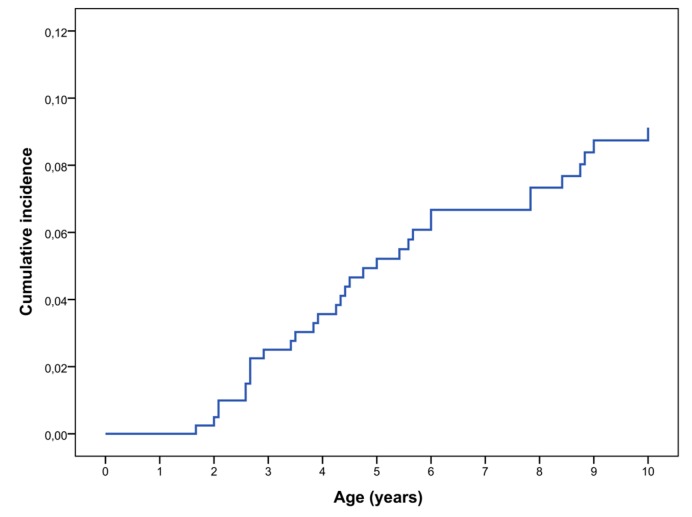
Cumulative incidence of symptomatic OPG in NF1 diagnosed patients in the first decade of life (N = 414) (Kaplan–Meier analysis).

We have re-evaluated these data and calculated the cumulative incidence of symptomatic OPG in the first decade of life; OPG was defined symptomatic if associated with any of the following anomalies: decreased visual acuity, ophthalmoscopic anomalies, optical coherence tomography (OCT) alterations, proptosis, precocious puberty, or other signs/symptom related to the tumor. In our cohort the rate of symptomatic OPG was 9.1% at 10 years of age (Kaplan–Meier analysis) (Figure 1). We excluded all the patients who underwent brain MRI scans for other clinical indications and were found to have OPG; anyway, none of these patients required any treatment during their follow up.

The prevalence of symptomatic OPG is comparable to that reported in other previous studies. However, we could not calculate the prevalence of asymptomatic OPG in our cohort since brain MRI was not systematically performed in all patients for screening purpose.

In the literature, the mean age at diagnosis ranges from 3 to 6 years [6,10,11,12,16], with estimates in the lower part of this range in studies with a systematic MRI screening program for NF1 patients [15,16,22,26]. OPGs rarely grow after 10 years of age [27] and few cases of adolescent or adult patients with a late onset or late-progressive OPG have been reported in the literature [25,28]. Additionally, OPG prevalence among adult patients was evaluated in few studies and was estimated to be approximately 5% [17,29].

Asymptomatic OPGs are prevalent in children with NF1 who are younger than 10 years and their frequency decreases with increasing age until adulthood [17]; conversely, the prevalence of symptomatic tumors (3% to 5% of patients with NF1) remains stable [16,17]. There are several hypotheses to explain the reduction of the prevalence of asymptomatic OPGs among adult patients: first of all, tumors may undergo spontaneous regression, as documented by several clinical reports [17,30,31,32]; additionally, since the histopathological examination of OPGs is rarely performed, there is the possibility that some of them are not true neoplasms.

Symptomatic OPG may present with decreased visual acuity, visual field deficits, optic disc swelling and/or disc pallor, proptosis, visual evoked potentials (VEPs) alterations, optical coherence tomography (OCT) abnormalities, strabismus, nystagmus, precious puberty, and various neurologic signs [6,20,33]. Signs and symptoms depend on the location of OPGs, which can arise anywhere along the optic pathway: intraorbital optic nerves, prechiasmatic optic nerves, chiasm, optic tracts and optic radiations [34]. Approximately 45 to 65% of OPGs involve only the prechiasmatic pathway, while 25% to 50% involve the chiasmatic region with or without the optic nerves; the optic tracts and radiations are affected (with or without the chiasmatic and/or prechiasmatic regions) in the remaining cases [6,15,16,17,25]. Symptomatic OPGs are associated with significant morbidity but there is no evidence that they are risk factors for premature death in NF1 patients [35].

Some prognostic factors associated with an increased risk of OPG clinical progression have been proposed. Females have been shown to lose vision and to require treatment in a higher proportion than males [15,24,36]; moreover, gliomas diagnosed in children younger than 2 years and older than 8–10 years are more aggressive than those presenting between 2–8 years of age [28,29,37,38]; finally, retrochiasmatic OPGs seems to have a more aggressive clinical behavior compared to prechiasmatic and chiasmatic ones [19,29,37]. However, conflicting results have been reported in the literature and currently no clear prognostic factors are available, making the clinical evolution of OPG unpredictable in the majority of cases [6,39].

### 2.2. Genotype-phenotype Correlations in OPG

The mutational spectrum at the *NF1* locus has been examined by a number of studies in order to evaluate the risk of OPG in NF1 asymptomatic patients. Despite the small number of recurrent mutations and the well-known intrafamilial variability in the expressivity of the disease, advances in molecular technologies in the last 20 years, with an increasing number of genotyped patients, have allowed to establish a growing number of genotype-phenotype correlations, with relevant consequences for the clinical management [40,41,42,43,44,45,46]. However, the variable timing, nature and location of *NF1* somatic mutations on the other allele significantly contribute to disease severity, making identification of such correlations challenging.

The relationship between OPG development and *NF1* genotype is still under debate. Some pathogenic variants in *NF1*, including the p.Met992del deletion detected in 170 individuals and the substitutions at codon 1809 identified in 98 patients, have never been reported in association with symptomatic OPG [41,43]; conversely, these tumors were found to be more prevalent in patients harboring missense variants affecting codons 844–848 [44]. Previous works focused on the risk of OPG in NF1 patients and reported conflicting results on the association between tumor development and mutation position, with some studies reporting an increased risk of OPG in patients harboring mutations in the 5′tertile of *NF1* (exons 1–21) [47,48], while others not confirming this finding [49]. In order to bypass limitations attributed to the small sample size, further adequately powered analyses on combined studies finally confirmed the correlation between the presence of 5′-end gene mutations and the likely of developing OPG (OR 2%, CI: 1.22–3.29; *p* = 0.006) [50,51,52]. Moreover, Xu and colleagues [51] elegantly showed that mutations clustering in specific regions of neurofibromin confer different risks of OPG: pathogenic variants correlate with a higher or lower risk of developing these tumors when located in the cysteine/serine-rich (CSRD, amino acids 543–909) or HEAT-like repeat region (HLR, amino acids 1825–2428) domains, respectively, despite the specific mutation type. These findings indicate that the *NF1* germline mutation might become a useful biomarker to predict OPG development and emphasize the importance of a better comprehension of the functions of neurofibromin. However, sensitivity and specificity of these correlations are currently not sufficient to provide an actual utility in the clinical setting [52], and further studies, evaluating other risk factors beyond the germline mutation, are warranted.

## 3. Screening and Diagnostic Protocol for OPG

### 3.1. The Role of Brain and Orbit MRI

Brain and orbit MRI is the gold standard neuroimaging examination for the diagnosis of OPG in symptomatic patients with NF1, but its role in the early detection of OPG in asymptomatic children is still controversial and subject of debate. In 1997, the NF1 Optic Pathway Glioma Task Force recommended against the use of screening MRI because there was no evidence it could improve the clinical outcome of patients, reducing the incidence of visual loss [11,13]. Several studies published in the following two decades confirmed the lack of significant differences in the outcome between symptomatic and asymptomatic patients at the time of OPG diagnosis; therefore, most of the protocols for the management of patients with NF1 continued not to recommend routine neuroradiological screening [5,6,16,20,27,53,54,55].

Conversely, few studies suggested that the early detection of OPG in young children may improve their clinical outcome [15,22]. Blazo et al. [22] observed that none out of 8 children diagnosed with an OPG by systematic MRI screening showed decreased vision (three additional children were lost to follow up); among these, five children had radiographically stable lesions with intact vision and three were treated with carboplatin and vincristine because of the progressive enlargement of chiasmal lesions without vision impairment. In contrast, decreased vision was documented in 5 out of 11 patients with known OPG or abnormal MRI at the first evaluation at the NF clinic (two additional children were lost to follow up); among these, four patients had undergone prior surgical intervention and two were treated with chemotherapy in response to demonstrated clinical and radiologic progression. However, Listernik and Charrow [54] noted that four out of five patients with visual impairment in the “unscreened” group presented with orbital tumors that had led to proptosis; they highlighted that such tumors are biologically different and are often characterized by rapid growth compared to their often quiescent chiasmal counterparts.

Prada et al. [15] observed that, among 149 patients with OPG, those with visual deficits at the time of diagnosis were more likely to develop visual decline despite therapy when compared with patients who were treated because of radiologic progression. The authors conclude that screening MRIs may lead to the early recognition and treatment of aggressive OPGs, with subsequent better visual outcomes.

However, in both the aforementioned studies [15,22], patients who retained a good visual function were treated after a radiological progression had been observed and did not show any relevant visual problems at the beginning of the therapy. Therefore, there is the possibility that normal vision could have been maintained even without any treatment.

Arguments supporting the current recommendations of not performing screening MRI are: (i) a single negative MRI does not exclude later onset of OPG; (ii) treatment is not required in the absence of progressive visual disturbance or other symptoms (including proptosis); (iii) periodic follow up MRI, often requiring sedation, needs to be carried out to detect the radiologic progression of the tumor; (iv) the vast majority of OPGs does not progress and does not require any treatment; (v) newly diagnosed OPG and follow up evaluations may raise parental anxiety; (vi) UBOs (unidentified bright objects) are frequent in children with NF1 but, although benign, may be difficult to differentiate from low-grade tumors; therefore, yearly follow-up brain imaging may be required after their detection.

On the other hand, Prada et al. [15] have suggested that screening MRI may be used to identify chiasmatic and postchiasmatic lesions, which have been proposed to have a high probability of progression and negative visual outcome; however, further studies are warranted to demonstrate whether the frequency of neuroimaging surveillance can be modified based on OPG location.

In addition, a role for neuroimaging screening needs to be considered for children in whom reliable visual assessment cannot be performed, because of the very young age or neurodevelopmental disorders [20].

Screening brain MRI is not recommended also in adult patients with NF1. In fact, despite the overall prevalence of brain tumors is higher than in the general population, the absolute risk does not support screening in asymptomatic patients. Moreover, OPGs arise during childhood and rarely progress after 10 years of age.

### 3.2. Ophthalmological Assessment

Recommendations for screening in patients with NF1 include annual eye examination in all children less than 8 years of age, and at least every 2 years until 18 years of age [20,27]. The main goal is to obtain a reliable age-appropriate assessment of visual acuity (VA). Anterior segment examination with slit lamp and ocular fundus examination by indirect ophthalmoscopy are also indicated. Conversely, visual field testing is most often unreliable in young children and optic disc pallor does not predict vision outcome [20,27]. Optical coherence tomography (OCT) has been recently introduced as an objective modality in the diagnosis and follow-up of NF1-associated OPG. This diagnostic tool measures retinal nerve fiber layer (RNFL) thickness, recording the secondary loss of optic nerve fibers, a reliable surrogate marker for visual loss in children with NF1 [56,57]. The use of this objective measure may circumvent some of the problems associated with an accurate vision assessment, especially in children with NF1 whose co-morbidity in terms of attention and cognitive disabilities have already been reported [20,27].

Once an OPG has been confirmed at MRI, the frequency of neuroimaging and ophthalmological assessment depends on the site of the tumor, the degree of visual impairment and the associated symptoms, as well as evidence of progressive disease [20]. In most experienced centers, eye examinations and VA testing are performed every 3 months for the first year after diagnosis, then every 6 months for 2 years until the age of 8, then annually until the age of 18, if stable [58].

#### 3.2.1. Visual Function Assessment

##### Visual Acuity Testing

Age-appropriate assessments of VA are critical for NF1-OPG surveillance and must be performed by an experienced (pediatric) ophthalmologist. VA assessment should always be performed as the first clinical test, before any other eye examinations such as optic disc evaluation and OCT scan [58,59]. Teller acuity cards (Figure S1), Lea symbols (Figure S2) or HOTV cards (Figure S3) and Snellen charts may be used in patients aged 0 to 2 years old, 2 to 6 years old and 6 to 15 years old, respectively. Patients aged 4 to 6 years old may undergo both Lea and Snellen test, starting with Lea, to compare the results and to accompany the transition of the growing child from one to the other test [59]. VA results should be compared to normal values for age (Table 1) [60,61]. VA assessment plays a primary role in determining disease progression and treatment decision, according to current guidelines. Thus, serial VA measurements are recommended to follow-up the affected patients. If a definite reduction in VA compared to age-based values is found, and it is not related to other ocular causes, MRI is recommended [27].

Since treatment is recommended in case of functionally significant tumor progression, vision abnormalities found at tumor presentation and considered chronic are not an indication for therapy. For the same reason, a blind eye without possibility of VA restoration, theoretically not requiring treatment, may suggest the necessity of careful follow-up of the fellow eye. In case of a blind eye, treatment may be also considered when significant proptosis, causing pain and corneal exposure, is present [58].

Serial MRI and assessments of visual function provide complementary information, mandatory for the management of OPG. Nevertheless, there is not a strict correlation between changes of VA and tumor volume, particularly after treatment [62]. From an ophthalmological point of view, the following findings have been proposed as criteria for clinical progression: (1) a 2-line change in Snellen, HOTV matching, or Lea matching VA compared with the previous examination or (2) a 2-line decline in Teller visual acuity [20].

When progressive visual loss is detected, other causes should be primarily excluded, such as refractive error, nonorganic visual loss, lack of cooperation, or ocular causes before the clinical findings can be attributed to tumor progression. Moreover, VA testing should be repeated within 1–2 weeks to confirm a presumptive tumor-related vision decrease before treatment decisions are taken [63].

Nonetheless, NF1 children with symptomatic OPG may have relevant ophthalmological abnormalities and moderate to severe impairment in cognitive functions, with attention difficulties, reducing the reliability of VA examination [56]. Moreover, a significant percentage of patients, particularly under the age of 5 years old, are unable to conclude VA testing [56,59]. Therefore, other functional and morphological tests have been proposed. None of them can, at present, be considered a full replacement to VA but they may be complementary to it and sometimes helpful in the treatment decision process.

##### Visual Field Testing

Visual field testing has been proposed as complementary to VA, since it may better define a VA loss in NF1-associated OPGs. Moreover, its results may be biased more than VA testing by attention deficits, typical of children, particularly if affected by NF1, being such test monotonous and time consuming [27,56]. Therefore, it is usually reserved to older or more collaborative children, to obtain a more precise definition and follow-up of the optic nerve related functional deficit [27,58]. Nevertheless, confrontation visual field, performed using finger counting or toys, may provide an initial indication for the clinician of the functional deficit in young children (<5 years old) [27,70]. Recently, defined reference values for visual field testing in children have been reported [71]. Unfortunately, in our experience, results of visual field testing are most often unreliable under the age of seven, and therefore we do not recommend performing this test routinely, reserving it for selected cases only.

##### Visual Evoked Potentials

The use of visual evoked potentials (VEPs), which measure cortical activity in response to a visual stimulus using electrodes over the scalp, has been proposed as a screening test [58]. Prolonged VEP latency or decreased amplitude may indicate damage to the visual pathway, identifying OPGs with high sensitivity (70–90%). However, specificity is low (58–69%) and VEP cannot distinguish between symptomatic and asymptomatic OPGs, giving results not corresponding to VA in a significant percentage of cases [58,62]. Moreover, each test takes a considerable amount of time (up to 30 min), making it bothersome in young children and requiring skilled clinical operators and clinicians to perform and interpret it. Therefore, its inapplicability in detecting functional worsening and monitor visual response to treatment does not support its use in standard practice [27,56,72].

##### Eye Movements Assessment

Strabismus and nystagmus, when caused by OPG, are also usually associated with VA loss. Nystagmus has been reported in 2–19% of OPG cases [71]. It may be present in chiasmatic or postchiasmatic OPG patients and may be horizontal or rotational, and asymmetric or monocular, also as presenting features. It may be associated with other neurological signs, proptosis and restrictive eye movement deficits [73,74].

OPG in children with NF1 is associated with an increased onset of strabismus, usually sensorial in nature. Although exotropia is the most common ocular misalignment associated with OPG, the direction of strabismus cannot be used as an accurate predictor for its presence [75].

Pupil reactions to light should be always performed in OPG patients and may be helpful in distinguishing an OPG-related VA loss from amblyopia and refractive errors, that are not associated with an afferent pupillary defect [71].

#### 3.2.2. Optical Coherence Tomography (OCT)

OCT is a non-invasive objective imaging modality, that allows for a precise measurement of retinal nerve fiber layer (RNFL) thickness in NF1-associated OPG. Currently, OCT is readily available in most secondary and tertiary ophthalmic referral centers, also in its most advanced and efficient modality, the Spectral Domain (SD) OCT. RNFL thickness measurement should be performed after pupil dilation, before optic disc evaluation, and after VA assessment, in order to increase its reliability. Peripapillary RNFL thickness (μm) measurements are automatically calculated by SD-OCT software, providing, in the majority of the devices, a global average and the average thickness for each peripapillary sector. The device also usually provides a pseudocolor map of different sectors, with colors corresponding to normal, borderline or pathological values compared to a normative database [56]. Because of the different databases used by different devices, all follow-up exams should be performed with the same device. Moreover, it should be considered that the inbuilt database is usually based on adult values (>18 years old). Therefore, younger patient’s values should be evaluated considering age-matched values, at least at baseline [56,76].

RNFL thickness proved to have a significant negative relationship with volumetric measurement of the entire anterior visual pathway and total brain volume, being a marker of damage to the visual pathway [77]. The use of this objective measure may circumvent some of the problems associated with accurately assessing VA (and other visual function tests), especially in NF1 children with comorbidities such as attention and cognitive disabilities [27]. RNFL analysis by OCT has been proven to correlate with VA and to have higher sensitivity, specificity, and positive and negative predictive values than both VA and optic disc evaluation. Moreover, OPG seems to cause RNFL loss (likely permanent) before becoming clinically manifest, suggesting a role for OCT in the early detection of optic nerve damage secondary to OPG [56].

**Figure 2:**
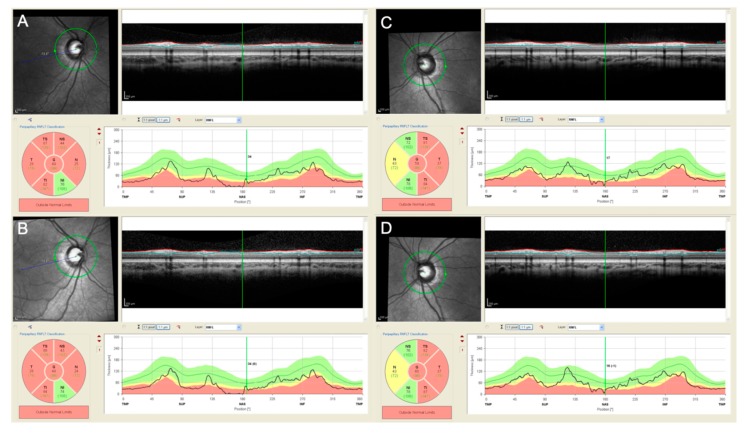
Peripapillary retinal nerve fiber layer (pRNFL) analysis performed in both eyes (**A**–**B** right eye, **C**–**D**, left eye) with spectral domain optical coherence tomography (SD-OCT, HRA+OCT Spectralis, Heidelberg Engineering, Heidelberg, Germany) in a 4 years old patient affected by neurofibromatosis type 1 (NF-1) and a optic chiasmatic-hypothalamic glioma. The patient was already treated with systemic chemotherapy and during the follow-up the visual acuity remained stable (0.52 logMAR in the right eye and 0.10 logMAR in the left eye). The pRNFL analysis performed in both eyes at the end of the chemotherapy (**A**,**C**) and 6 months after (**B**,**D**) confirm the stability of the pRNFL thickness during time.

OCT data acquisition requires some collaboration from the patient. However, acquiring a reliable scan requires only a few seconds of fixation. Therefore, although an interindividual variability exists, reliable results may be obtained even in young children. OCT has the advantage to provide automatically generated numeric values and its follow-up modality can be used to obtain a precise, automatic, operator-independent, numeric evaluation of any changes in RNFL thickness, with a precision of few microns [56] (Figure 2). Therefore, RNFL thickness has been suggested as a very reliable surrogate marker of VA in young children where VA testing is unpredictable. The best balanced global cut-off values (e.g., 76 μm for the global average) have been proposed to discriminate false-positive VA results (Table 2). RNFL thickness values above the cut-off could reassure clinicians, patient and parents that the tumor, despite being present, is not causing significant VA reduction at that time, even when reliable VA measure is unavailable. This may allow to defer the treatment until direct evidences of VA impairment secondary to the tumor presence appears [57].

At follow-up, RNFL thickness reduction greater than 10% (in one or more quadrants or global average) has been proven to be highly predictive of new vision loss in patients affected by OPG. Conversely, a stable RNFL thickness in serial re-examinations has shown to be highly predictive of stable vision [78]. However, in patients with normal RNFL thickness a greater amount of RNFL thinning may be necessary to cause significant visual loss, compared to patients with an already thinned RNFL [78].

Other OCT scan tests have been proposed, such as the macular map for the analysis of the ganglion cell layer, which has been proven to correlate with VA. However, the evaluation of the peripapillary RNFL thickness currently appears as the most significant and quickest analysis to be performed, also compared to the macular map scan that requires more time and more fixation ability. Finally, even if a handled OCT usable at bed has been produced, these data can be obtained without patient sedation in most children [57,79,80].

SD-OCT devices also perform near-infrared reflectance (NIR) imaging, that is able to detect choroidal nodules, characteristic of NF1 patients and currently under evaluation to be included as a new NF1 diagnostic criterium. Therefore, the combined use of SD-OCT and NIR imaging has the advantage of evaluating two relevant clinical features in NF1 patients with OPG and should be considered as a mandatory, routine procedure in these patients [81,82].

Recently a new modality of OCT, the OCT angiography, has been applied to NF1 patients with OPG. This non-invasive imaging modality allows for a precise visualization of retinal and optic disc vascularization without dye injection. Even though its use is currently limited to research purposes, it may provide new insights into the complex interrelations between vascular and neural retinal changes secondary to OPG [83,84].

#### 3.2.3. Optic Disc Evaluation

Optic disc evaluation should be performed by an experienced pediatric ophthalmologist using indirect ophthalmoscopy, after mydriasis and after VA and RNFL assessment. Signs of optic disc swelling, pallor, atrophy, asymmetry, or excavation should be reported [56]. Their incidence in OPG patients varies widely among studies and they may be associated (or not) with VA loss, but do not predict it. Therefore, their appearance alone should not constitute evidence of clinical progression nor an indication for treatment [27,63,70].

## 4. Preclinical Models of OPG

The pathogenetic mechanisms at the basis of OPG development in NF1 are not completely understood, thus reducing the possibility to develop effective treatments. The main limitations for the study of the molecular pathways leading to OPG have been the paucity of biological samples, given the low rate of surgery, and the difficulty to obtain patient-derived xenograft-models or cell lines from these tumors [85]. However, over the last few years distinct strategies have been used to investigate gliomagenesis.

No single model has been found to fully recapitulate the human tumor phenotype, but the combination of different research approaches (ranging from the employment of animal models with increasing complexity to the molecular characterization of human brain tumors and the use of engineered patient’s modified iPSC cells) has allowed to generate a fertile ground for future translational research projects. Hopefully, results obtained from these models will be applied in the clinical setting in order to stratify patients, to better define the prognosis and to promote novel clinical trials with biological drugs or other innovative therapies.

A number of preclinical models of NF1 have been developed, but only some of them are characterized by the development of OPG. Notably, most of our understanding of the biology of these tumors of the central nervous system derives from engineered NF1 mice. Since germline ablation of both copies of *NF1* is embryonically lethal in rodents [86], Cre-*lox*P technology has been employed to conditionally inactivate this tumor suppressor gene in specific time windows during embryogenesis and/or in different neuroglial progenitors, yielding a series of distinct engineered mice with NF1 developing OPG [87,88].

These murine models allowed a better comprehension of gliomagenesis: somatic *Nf1* loss induced by Cre expression driven by different cell-specific promoters or induced by tamoxifen during embryogenesis, defined neural stem/progenitor cells (NPCs) and the more committed oligodendrocyte precursor cells (OPCs) as potential cell(s) of origin of OPG with latency of tumor development of 3 and 6 months, respectively [87,89,90]. Furthermore, these engineered models of NF1 were crucial to confirming the role of RAS hyperactivation in the development of OPG, providing the biological rationale to test a series of compounds able to counteract the effectors downstream to RAS: the RAS-MEK-ERK and the PI3K-AKT-mTOR signaling pathways are up-regulated in tumor cells [91,92,93], whereas the adenylyl cyclase-mediated cyclic AMP is associated with decreased levels of cAMP [94]. However, some of the drugs acting on these pathways (including the mTOR inhibitor Rapamycin) are active in mice only during therapy, with recovery of tumor growth after treatment suspension [85].

Additionally, murine models of NF1-associated OPG have provided relevant information regarding the role of microenvironment on tumor growth, and particularly of microglia, immune system-like non-neoplastic cells which, unlike glioma cells that undergo loss of heterozygosity (NF1^−/−^), maintain the heterozygous state (NF1^+/−^). A series of studies elegantly showed that these cells are required to sustain tumor growth, since loss of *NF1* in neuroglial progenitor cells alone does not result in gliomagenesis [87,88]. Remarkably, interaction between stromal cells (mainly microglia) and tumor cells is mediated by soluble factors that might be targetable: it has been shown that both genetic and pharmacological inhibition of microglia function is able to reduce OPG growth [95,96,97]. Microglial gliomagens include chemokines, growth factors and inflammatory mediators; among a series of candidate molecules, Ccl5 was found enriched in murine OPG and treatment with Ccl5 neutralizing antibodies reduced tumor growth in mice [97]. Furthermore, Guo and coauthors demonstrated that OPG variability in mouse relies on the ability of the tumor to secrete specific molecules that recruit T lymphocytes and microglia, rather than on the intrinsic growth ability of the tumor [98]. T lymphocytes and microglia are required to establish a supportive tumor environment, as shown by studies in which their content correlate with tumor grade, but further studies are ongoing to define how they affect tumor growth.

Although translation from mouse models to patients remains challenging, these preclinical studies provide the bases to outline clinical trials in NF1 children. An effective treatment undoubtedly requires a better definition of tumor molecular signatures and probably needs a multiple-stage intervention, targeting both glioma cells and stromal non-neoplastic cells.

Besides, preclinical models have been fundamental to elucidating that vision loss due to NF1-associated OPG depends on retinal ganglion cell (RGC) death. Although no clinical trials addressing this pathogenetic pathways are currently available, strategies promoting RGC survival are under investigation in mouse [99].

Recently, other mammalian models of NF1 have been generated, including the minipig that develops OPG in a proportion similar to humans. Considering the higher similarities between humans and pigs compared to rodents in many physiological functions (including drug metabolism), this new model of NF1 offers an unprecedented opportunity to study the natural history of these tumors and to test novel treatments [100].

Despite the progress in our understanding of OPG pathogenesis, only few molecules proficiently tested in mouse models (including selumetinib) have been found to be effective in clinical trials, highlighting the considerable differences among species, as for instance the lower microglia content in rodents compared with humans. To overcome these limitations, mouse models are now supported by the use of induced pluripotent stem cells (iPSCs) derived from NF1 patients [101]: human microglia-like cells have been successfully reprogrammed from NF1 iPSCs [102], providing the bases for generating in vitro co-cultures of the two key players of OPG development.

Beyond its involvement in NF1, the *NF1* gene behaves as a tumor suppressor in a number of sporadic malignancies [103]. A recent study compared the molecular signatures of NF1-associated glioma (including OPG) with their sporadic counterpart, allowing to cluster low- and high-grade lesions and pinpointing distinct biological features that might have relevant implications for the development of novel treatments [104]. Although this study obviously analyzed only those neoplasms requiring surgery (that is not usually the choice of treatment for these tumors) and included only a small proportion of OPGs, it highlights the following important issues: first, the heterogeneity of the molecular landscape of NF1-associated gliomas; second, the presence of lymphocytic infiltrates in a fraction of these tumors, thus indicating a potential application of immunotherapy.

## 5. Oncologic Treatment of Children with NF1-OPG

### 5.1. Standard Treatment

Systemic cytotoxic chemotherapy has been the mainstay of treatment in children with progressive NF1-OPG, with the combination of vincristine/carboplatin (VC) as the most used, and effective regimen, leading to an excellent overall survival, and satisfactory long-term tumor control.

However, in view of the unpredictable nature of NF1-OPG, anti-neoplastic treatment is only recommended, after an initial *watch and wait* period, in case of documented clinical and/or radiological progression, with the former criteria defined as visual acuity loss of at least 0.2 logMAR units or new visual field loss appearance. [33,58,105].

Other risk factors, as pre-existing threat to bilateral vision, as well as chiasmatic/post-chiasmatic OPG involvement, or progressive tumor growth, influence the overall decision, in favor of starting treatment. It is less clear whether young age at OPG diagnosis may constitute an independent prognostic factor for OPG progression, but young children (<1–2 years), may also need treatment, especially in the presence of abnormal vision for age, or unreliable visual testing [33,58].

Treatment duration may vary between 12 and 18 months, according to two largest prospective trials in US (12 in COG A9952), and Europe (18 in SIOP-LGG2004), but with similar results in terms of 5-yr progression-free survival (PFS) of 69% ± 4%, and 71% ± 6%, respectively [20,106].

Overall, VC is a well-tolerated regimen, with manageable hematological toxicity, and peripheral neuropathy and autonomic neuropathy, are the most frequent side effects with vincristine. Carboplatin related renal or ototoxicity are rare and mild, but need a regular monitoring throughout the treatment. Allergy to carboplatin can also occur with increased frequency, especially after the first months of treatment, and requires longer infusion, pre-medication, or change to alternative regimens, in case of severe or repeated reactions. There are very few, if any, long-term side effects with this regimen. However, chemotherapy-induced structural changes in cerebral white matter have been recently described in children with NF1-associated glioma treated with low-intensity chemotherapies [107]. These neuroradiological findings require long-term follow-up studies to clarify their functional significance. Despite evidence of good response to chemotherapy, children with NF1-OPG have a variable functional outcome, particularly in term of vision, with lack of a clear clinic-radiologic correlation. In fact, ≤1/3 of children with NF1-OPG improve their vision, while ≥1/3 have a stable, and ≈1/3 a worse visual acuity (VA) during or after chemotherapy. Predictive factors for visual outcome are not defined yet, but age after 2 years and no post-chiasmatic involvement are often associated with VA improvement with chemo [19,108,109,110,111]. Moreover, despite chemotherapy being able to stabilize vision in about 2/3 of patient, it is of further concern that about half of these patients started treatment with an already compromised vision in one or both eyes.

Other chemotherapy regimens might have comparable efficacy, including weekly vinblastine (5-yr PFS = 70–75%), a cheap and widely available drug, which is now offered as standard of care in some countries, either upfront or at relapse, in view of its favorable toxicity profile, compared to the VC regimen [112,113]. The efficacy and toxicity of vinblastine, alone or in combination with non-cytotoxic agents as nilotinib (a multi TKI possibly targeting tumor microenviroment) or anti-VEGF (Bevacizumab), are being tested in two randomized phase II trials in children with progressive newly diagnosed NF1-LGG. Results are expected soon and might possibly extend the treatment armamentarium for these children.

Consistent data from various retrospective series indicate that Bevacizumab, alone or in combination, can lead to visual improvement as well as radiological response in a significant proportion (50–70%) of children with multiply relapsed OPG with or without NF1 [114].

While bevacizumab has proven to be effective and with a reasonable toxicity including systemic hypertension, and proteinuria, when treatment duration extends beyond 12 months [115], more than 40% children recur within few months after drug stop. Its effect is not durable and its role needs to be reconsidered in view of the high costs.

### 5.2. MEK Inhibitors

Recent data on preclinical models have confirmed the constitutional activation of the MAPK/ERK/MEK pathway as a direct consequence of NF1-associated loss of neurofibromin function, prompting recent clinical trials employing inhibitors of mitogen-activated protein kinase (MEK) for the treatment children with NF1-associated tumors, including OPG [92]. Selumetinib (AZD6244) is a potent, selective, orally available, non-ATP competitive small molecule inhibitor of MEK-1/2, which has shown in vivo significant growth inhibition in NF1-associated plexiform neurofibromas [116]. A recently published phase II clinical trial with the use of selumetinib in relapsed LGG, including NF1-associated OPG, showed that ten (40%) of 25 NF1-LGG patients achieved a sustained partial response after a median follow-up of 48 months, and only one patient progressed while on therapy. The 2-year PFS was 96 ± 4% [115].

A prospective randomized phase 3 study, with the aim of comparing the efficacy of selumetinib vs. carboplatin-vincristine in terms of event-free survival (EFS) and visual function (primarily assessed by Teller Acuity Cards), in previously untreated progressive NF1-OPG, might be soon launched as a result of a joint collaboration between pharma, US (COG), as well as European (SIOP) brain tumor experts.

Furthermore, a phase I/II trial (INSPECT) will further assess dosing, toxicity, and preliminary efficacy of an intermittent on/off schedule of selumetinib in children with relapsed NF1-OPG in selected centers.

While selumetinib is the most advanced drug in terms of efficacy and toxicity data in the NF1 population, other MEK inhibitors (MEKi) such as trametinib, cobimetinib, or binimetinib, are currently being tested in early phase clinical trials, or as compassionate use, in children with OPG, with or without NF1, and may be also considered.

Skin toxicity is the most commonly observed side effect from MEKi, and it is more frequent in adolescent and post pubertal children, as acneiform, follicular rash or paronychia. While toxicity is usually mild and reversible, it requires prompt diagnosis and timely treatment to avoid the need for therapy interruption. Prevention of acneiform rash includes the regular use of thick emollient creams. Other less frequent side effects include diarrhea, edema, fatigue, hypertension, constipation, pain, pneumonitis, as well as asymptomatic CPK elevation. Retinal findings (e.g., retinal pigment epithelial detachment (RPED) due to subretinal fluid accumulation or retinal vein occlusion (RVO) are both associated with MEKi [117,118]. If RPED is diagnosed, treatment with MEKi should be discontinued until resolution, or permanently if RVO occurs. Both RPED and RVO should be monitored with fundoscopic exam and/or OCT.

Although MEKi proved to be effective in NF1-associated tumors including OPG, several questions remain unanswered, including the possible occurrence of resistance mechanisms, the effect durability, how to overcome and most importantly, the long term side effects, especially in comparison with current chemotherapy.

### 5.3. Other Potential Treatments for NF1-OPG

A randomized trial from Falsini et al. [119], showed that the regular use of nerve growth factor (NGF) eyedrops could possibly improve visual fields, VEPs and/or other electrophysiological parameters in 13 patients with NF1-OPG who received experimental NGF after the end of chemotherapy. A phase II trial will hopefully confirm the potential benefit of this innovative approach, with potentially very few, if any, systemic side effects.

Genetically engineered mouse (GEM) models with NF1 associated tumors showed that pharmacologic elevation of cAMP, by the use of PDE4 inhibitors (rolipram) resulted in a dramatically reduction of optic glioma growth. Unfortunately, these exciting preclinical data have not undergone further translation into clinical trials yet [120].

## 6. Conclusions

The current treatment for progressive OPG in NF1, based on systemic chemotherapy is not optimal, at least in terms of visual outcome. [120] New and more effective strategies should include objective biomarkers of vision loss to better evaluate *who and when* children with NF1-OPG need treatment, and to assess treatment response particularly in terms of functional outcome. New insight into MRI volumetric measures may further help to stratify the risk of visual loss from NF1-OPG by predicting axonal degeneration, as initially described by Avery et al. [77]. The next generation of NF1-OPG trials incorporating visual outcome as a primary outcome will likely provide more robust and evidence-based data on the role of these new agents for the treatment of NF1-OPG.

## Figures and Tables

**Table 1:** Visual acuity norms for pediatric populations by test type *.

Test	Authors	Age (Years)	Mean Visual Acuity	logMAR Equivalent	Approximate Snellen Fraction
Teller acuity cards	Mayer et al. [64]	(1 month)	1 cycle per degree	1.5	6/180
		(6 months)	6 cycles per degree	0.70	6/30
		4	25 cycles per degree	0.07	6/7
	Hargdon et al. [65]	5–6	24.5 cycles per degree	0.10	6/7.5
Single Lea symbol	Becker et al. [66]	2–6	0.88 Snellen decimal	0.07	6/7
Crowded Lea symbols	Becker et al. [66]	2–6	0.74 Snellen decimal	0.12	6/8
	Chen et al. [67]	4.5–8.5		0.08 ± 0.09	6/7.5
HOTV crowded	Drover et al. [68]	3–4		0.08	6/7.5
	Drover et al. [68]	5		0.03	6/6
	Drover et al. [68]	6		−0.03	6/6
	Pan et al. [69]	3–4		0.17 ± 0.13	6/9
		4–6		0.08 ± 0.11	6/7.5

* Modified from Anstice et al. [60]. Mean (± standard deviation where data were available) values for visual acuity are shown in the units published, as well as logMAR and Snellen equivalents, where appropriate.

**Table 2:** Best balanced and most sensitive cut-off values of each RNFL analyzed sector predicting visual acuity in OPG pediatric patients *.

RNFL Thickness	Cut-off	SE	SP	PPV	NPP
RNFL thickness (G)					
Most sensitive	88 µm	100.0%	55.8%	54.8%	100.0%
Best balanced	76 µm	91.3%	76.7%	67.7%	94.3%
RNFL thickness (T)					
Most sensitive	59 µm	100.0%	60.5%	57.5%	100.0%
Best balanced	49 µm	87.0%	76.7%	66.7%	91.7%
RNFL thickness (S)					
Most sensitive	115 µm	100.0%	41.9%	47.9%	100.0%
Best balanced	95 µm	87.0%	81.4%	71.4%	92.1%
RNFL thickness (N)					
Most sensitive	111 µm	100.0%	2.3%	35.4%	100.0%
Best balanced	54 µm	78.3%	72.1%	60.0%	86.1%
RNFL thickness (I)					
Most sensitive	117 µm	100.0%	51.2%	52.3%	100.0%
Best balanced	99 µm	87.0%	79.1%	69.0%	91.9%

* Modified from Parrozzani et al. [57] SE: specificity; SP: sensibility; PPV: positive predictive value; NPV: negative predictive value: retinal nerve fiber layer; G: Global; T: temporal; S: superior; N: nasal; I: Inferior.

## Supplementary Materials

The following are available online at https://www.mdpi.com/2072-6694/11/11/1790/s1, Figure S1: Photograph of 4 Teller cards, Figure S2: The Lea symbols chart, Figure S3: The HOTV symbols chart.
