# Supplementary material for: Optic Pathway Glioma in Type 1 Neurofibromatosis: Review of Its Pathogenesis, Diagnostic Assessment, and Treatment Recommendations
Source: Cancers (Basel). 2019 Nov 14;11(11):1790. doi: 10.3390/cancers11111790 (PMC6896195; doi:10.3390/cancers11111790)

Figure S1. Photograph of 4 Teller cards. Considering a test distance of 38 cm, the first card from the top corresponds to 20/45 Snellen equivalent or 13 cycles per degree, the second to 20/66 Snellen equivalent or 9.1 cycles per degree, the third to 20/180 Snellen equivalent or 3.2 cycles per degree and the firth to 20/1400 Snellen equivalent or 0.44 cycles per degree.

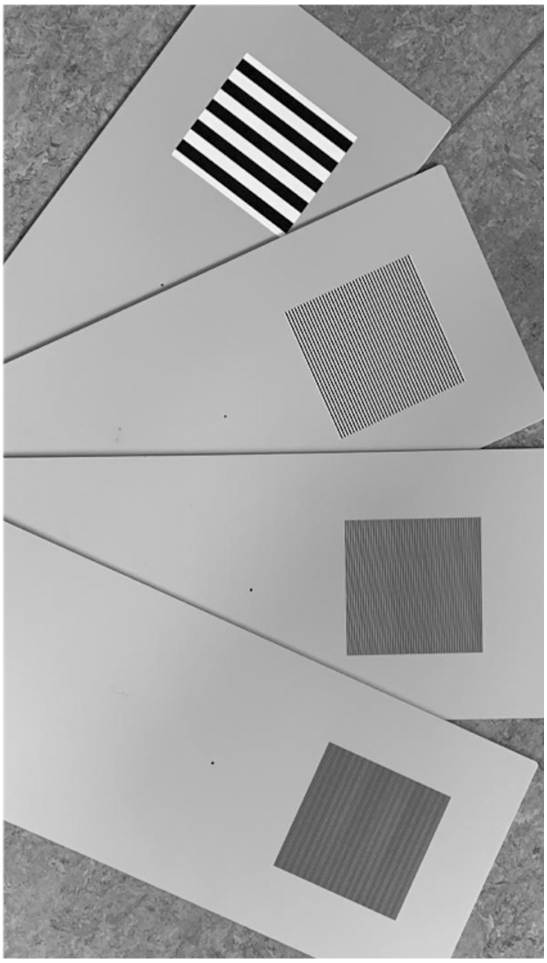

Figure S2. The Lea symbols chart.

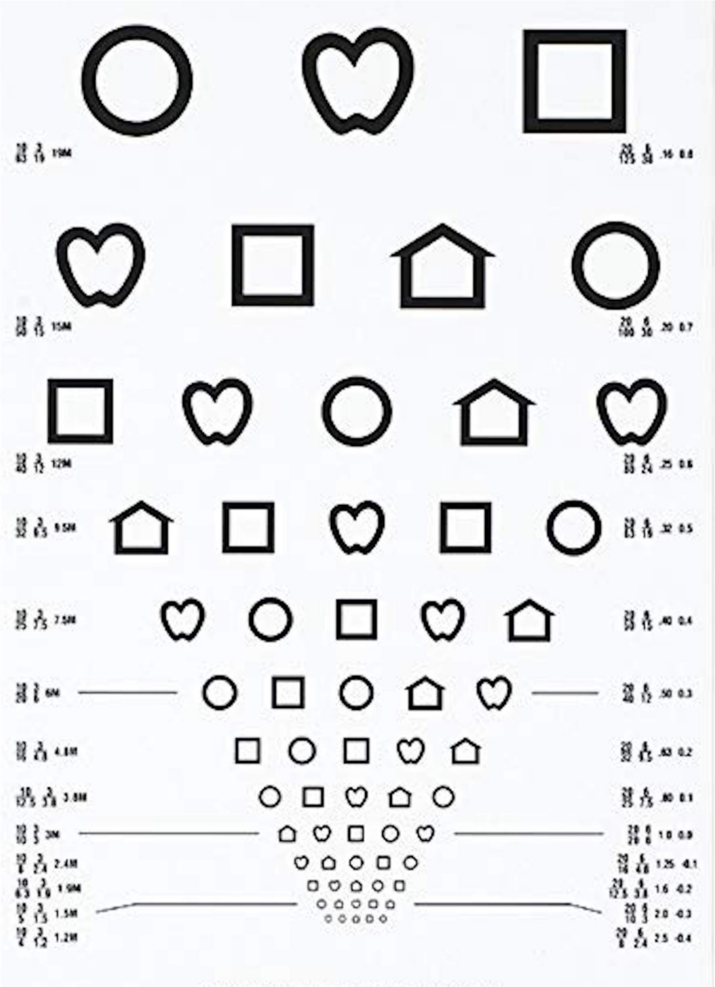

Figure S3. The HOTV symbols chart.

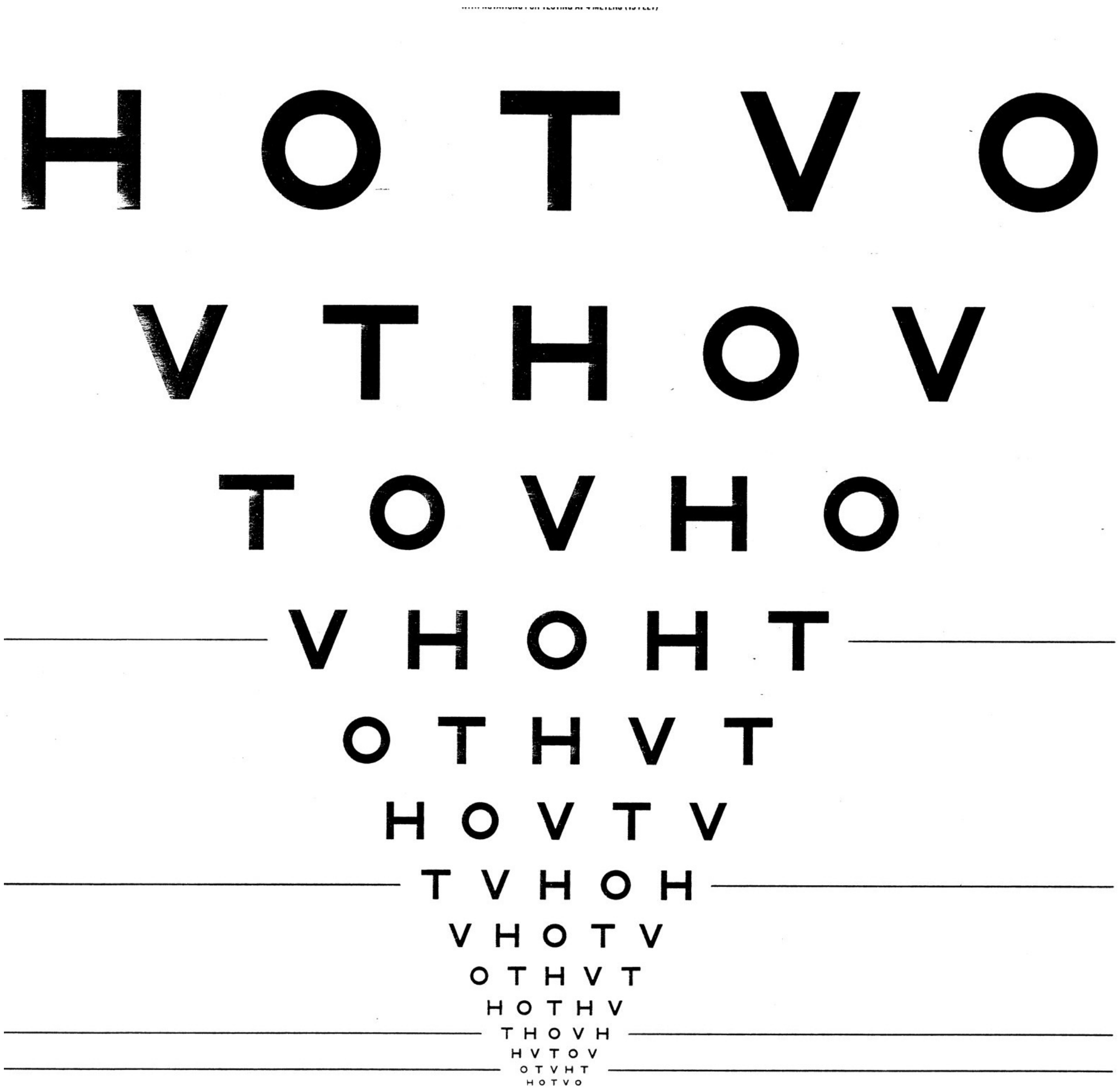

Supplement: Supplementary file 1 [file cancers-11-01790-s001.zip › Supplementary Materials.pdf]
